# Training needs assessment of health care professionals in a developing country: the example of Saint Lucia

**DOI:** 10.1186/s12909-016-0638-9

**Published:** 2016-04-16

**Authors:** Janice Gaspard, Che-Ming Yang

**Affiliations:** The Epidemiology Unit of the Ministry of Health, 2nd Floor Sir Stanislaus James Building, Waterfront, Castries, Saint Lucia; Shuang Ho Hospital, Taipei Medical University; School of Health Care Administration, College of Management, Taipei Medical University, 250 Wu-Hsing Street, 110 Taipei, Taiwan (R.O.C.)

**Keywords:** Continuing education, Continuing professional education, Training needs assessment

## Abstract

**Background:**

Continuing education (CE) is crucial for quality improvement in health care. The needs assessment of CE helps ensure effectiveness. However, such an assessment necessitates certain techniques that are unfamiliar to health care communities in developing countries. This study identifies the needs of providing CE to health care personnel in Saint Lucia.

**Methods:**

This study was designed as a questionnaire survey to investigate the demographics, training needs, and preferred approaches to improve performance of the target population. The study population included the health care professionals of major public health care facilities in Saint Lucia. We used the World Health Organization–adopted Hennessy Hicks Training Needs Analysis Questionnaire, a self-reported close-ended structured questionnaire with a core set of 30 items. These items refer to tasks that are central to the role of health care professionals and are categorized into six superordinate categories: research/audit, communication/teamwork, clinical skills, administrative, managerial/supervisory, and continuing professional education.

**Results:**

In total, 208 questionnaires were distributed; the response rate was 66.8 %, and most respondents were nurses. The need for continuing professional education was rated the highest priority, followed by research/audit activities. The evidence suggests that most respondents required training in communication skills, management, clinical skills, and research methods.

**Conclusions:**

Providing training according to the needs is vital, particularly in developing countries. The present research methodology and findings offer perspectives on how to conduct needs assessment and offer reference points for developing countries whose background and health care environment are similar to those of Saint Lucia.

**Electronic supplementary material:**

The online version of this article (doi:10.1186/s12909-016-0638-9) contains supplementary material, which is available to authorized users.

## Background

Saint Lucia is a small developing country located in the Eastern Caribbean and has a population of approximately 166 526 persons [1]. It covers a land area 42 km long and 22 km wide and has a shoreline of 155.7 km [2]. In Saint Lucia, the average life expectancy at birth was 77.22 years in 2013, and the hospital bed density was 1.6 beds/1000 persons [3]. Health care spending was estimated to be 7.2 % of the gross domestic product in 2011 [3] and was provided primarily through publicly funded establishments. The Ministry of Health (MOH) is responsible for managing all publicly funded health care initiatives.

Continuing education (CE) is crucial for quality improvement in health care. However, CE is often provided without much planning [4], which happens in developed countries [5] and developing countries. A needs assessment of CE of all the involved parties is crucial to ensure CE effectiveness [5].

Contrasted with developed countries, healthcare professionals in low-resource countries oftentimes do not have the obligation to demonstrate ongoing education or competence; Past research had found low levels of provider training and huge quality gaps in less developed countries [6].

The Health Review Commission Report of 2004 [7] indicates no evidence of a training needs assessment conducted for health care professionals in Saint Lucia. The report recommended that the MOH conduct a training needs assessment and that its results be used to plot career development paths. Since this report, no further studies have been conducted to assist in implementing the report recommendations.

Many studies [8–11] have recommended needs assessment before implementing any educational program; therefore, such an assessment is necessary in this setting to guide the policy development of training for all levels of health care personnel. However, such an assessment necessitates certain techniques that are unfamiliar to health care communities in developing countries.

This study identified the needs of providing CE to health care personnel in Saint Lucia. The results are expected to provide insight to policy makers, financers, and health care organizations for guiding training planning and funding allocation required for continuing professional education. In addition, this study offered perspectives on how to conduct training needs assessment and serves as a reference for developing countries whose environments are similar to that of Saint Lucia.

## Methods

### Study design

This study was designed as a questionnaire survey to investigate the demographics, training needs and preferred approaches to improve the performance of the target population. The study population included health care professionals of major public health care facilities in Saint Lucia. Ethical approval was obtained from the Saint Lucia Medical and Dental Council on August 9, 2011.

### Instrument

This study used the Hennessy Hicks Training Needs Analysis Questionnaire (see Additional file 1), which has been psychometrically tested for validity and reliability [12]. It is adopted by the World Health Organization as a training assessment tool [12]. The main part of the questionnaire comprises a core set of 30 items that was minimally modified to meet the requirements of this study without compromising its psychometric properties.

All 30 items refer to tasks that are central to the role of health care professionals and are categorized into six super ordinate categories: research/audit, communication/teamwork, clinical skills, administrative, managerial/supervisory and continuing professional education (CPE). Respondents rate each item on a seven-point scale according to two criteria: how critical the task is to the successful performance of the respondent’s job and how well the respondent is currently performing the task. The ratings for the first criterion provide an overall occupational profile of the respondent’s job, and those for the second criterion provide an index of the skill level or performance [13].

This instrument allows for measuring training needs within broad categories as well as enables comparisons between categories. Therefore, each category can be used independently or in combination with other categories to obtain the required information. The instrument is semi-opaque meaning that the respondents are less likely to be able to distort their responses; therefore, the obtained data would more accurately reflect the training requirements [14]. Thus, the instrument has an advantage over other similar instruments. Furthermore, the instrument has been extensively used by health care professionals for various reasons: for instance, to identify the training needs of primary care nurses and community and hospital-based nurses [15]. It has also been used by allied health care professionals in cross-cultural studies of health care practitioners and to identify continuing development needs of specialists [16]. In most of these studies, this instrument has been customized according to the target objectives, as in this study.

Simple subtraction was performed for each individual questionnaire item by subtracting the performance score from the importance score, thus yielding a difference score that reflected the extent of training need. A comparison of the two ratings on any item provides an assessment of the associated training need. The results suggest that tasks considered to be highly crucial but not well performed require training, whereas items with similar scores for criticality and performance require little training.

The training needs were prioritized to identify those requiring maximum development, which is highly relevant where training and education budgets are restricted. The reliability of comparing importance and performance scores for identifying training needs has been well established. In addition, the questionnaire has a section for biographical and occupational information, thus providing the opportunity of classifying the sample into different groups for comparative analyses.

### Data collection procedure

The questionnaires were distributed and collected during July 2011– August 2011. Data were collected from 10 major public health care facilities in Saint Lucia, namely Victoria Hospital, St. Jude Hospital, Gros Islet Poly Clinic, Babonneau Health Center, Dennery Hospital, Anse la Raye Health Center, Castries Health Center, Soufriere Hospital, Senior Citizens Home (Comfort Bay Senior Citizens’ Home), and St. Lucia National Mental Wellness Center. At least one health care center was included from each of the health districts.

Stratified convenience sampling was used. The on-duty personnel from the various health departments were provided a self-completion questionnaire, which were returned to the data collectors after completion on the same day. The questionnaires were completed by the interviewers for those respondents who did not wish to complete it themselves. Each department was assigned a quota on the basis of the number of employees.

The respondents of this study belonged to various health disciplines in Saint Lucia and included nurses, physicians, radiology technicians, medical technologists, other allied health care professionals, and administrative staff. All health care professionals who were full-time employees at any of the government-owned health facilities and were willing to participate were included in the study. Health care personnel employed by private entities were excluded from the study.

## Results

In all, 208 questionnaires were distributed; the response rate was 66.8 %, which was satisfactory when compared with the rates reported in similar studies on health care professionals that used the same tool [17–19].

In Saint Lucia, most health care workers are women. Accordingly, most respondents in this study were females (Table 1). Most respondents belonged to the 29–39 years age group, as was the case in related studies [17, 18]. Younger professionals were found to require more training than did professionals from other age groups. This finding is consistent with those of other studies that suggest that the younger generation typically has higher career aspirations and is therefore more likely to undertake further training.

**Table 1:** Descriptive statistics for demographic and control variables

	Number	Percent	Mean	SD
Age				
≤28	37	30.1	35.42	10.17
29–39	53	43.1		
≥40	33	26.8		
Sex				
Female	110	79.1		
Male	29	20.9		
Profession				
Physician	22	15.8		
Pharmacist	8	5.8		
Laboratory Technician	7	5.0		
Administration	14	10.1		
Nurse	78	56.1		
Radiology Technician	3	2.2		
Physiotherapist	4	2.9		
Other	3	2.2		
Number of years since qualified				
<1 year	15	10.8	3.63	1.745
1–3 years	32	23.0		
4–6 years	27	19.4		
7–9 years	12	8.6		
10–15 years	21	15.1		
>15 years	32	23.0		
Place of work				
Acute general hospitals	111	80.43		
District hospitals	6	4.35		
Primary healthcare centers	10	7.25		
Hospice care	2	1.45		
Specialized care	9	6.52		
Previous work-related training				
No	36	26.7		
Yes	99	73.3		
Number of sessions				
None	38	29.2	2.96	1.343
1	5	3.8		
2	11	8.5		
>2	76	58.5		
Educational level				
Diploma	23	17.4		
Associate’s degree	53	40.2		
Bachelor’s degree	44	33.3		
Master’s degree	8	6.1		
PhD	0	0		
Other post graduate certificate	4	3.0		
Management/Supervisory position				
No	80	58.4		
Yes	57	41.6		

Most respondents were nurses (56.1 %) because they tend to be more receptive to answering and assisting in data collection and participating in programs for further professional growth compared with physicians (15.8 %). Typically, physicians are reluctant to participate in such research.

An equal number of respondents (23 %) had 1–3 years and more than 15 years of work experience; thus, these two groups formed the majority when the respondents were classified by seniority.

A considerable number of respondents were not at any management or supervisory position at the time of the survey (58.4 %). Similar to any health care organization, the general staff outnumber the managerial staff.

Most respondents worked for acute general hospitals. As anticipated, Victoria Hospital accounted for the highest number of respondents (56.5 %) because it is the largest health care facility in the country and employs the most health care personnel.

Of the surveyed health care professionals, over 70 % had undergone previous work-related training; of these, 58.5 % of them had undergone more than 2 sessions. This indicates that some level of training occurs at the institutions.

**Fig. 1:**
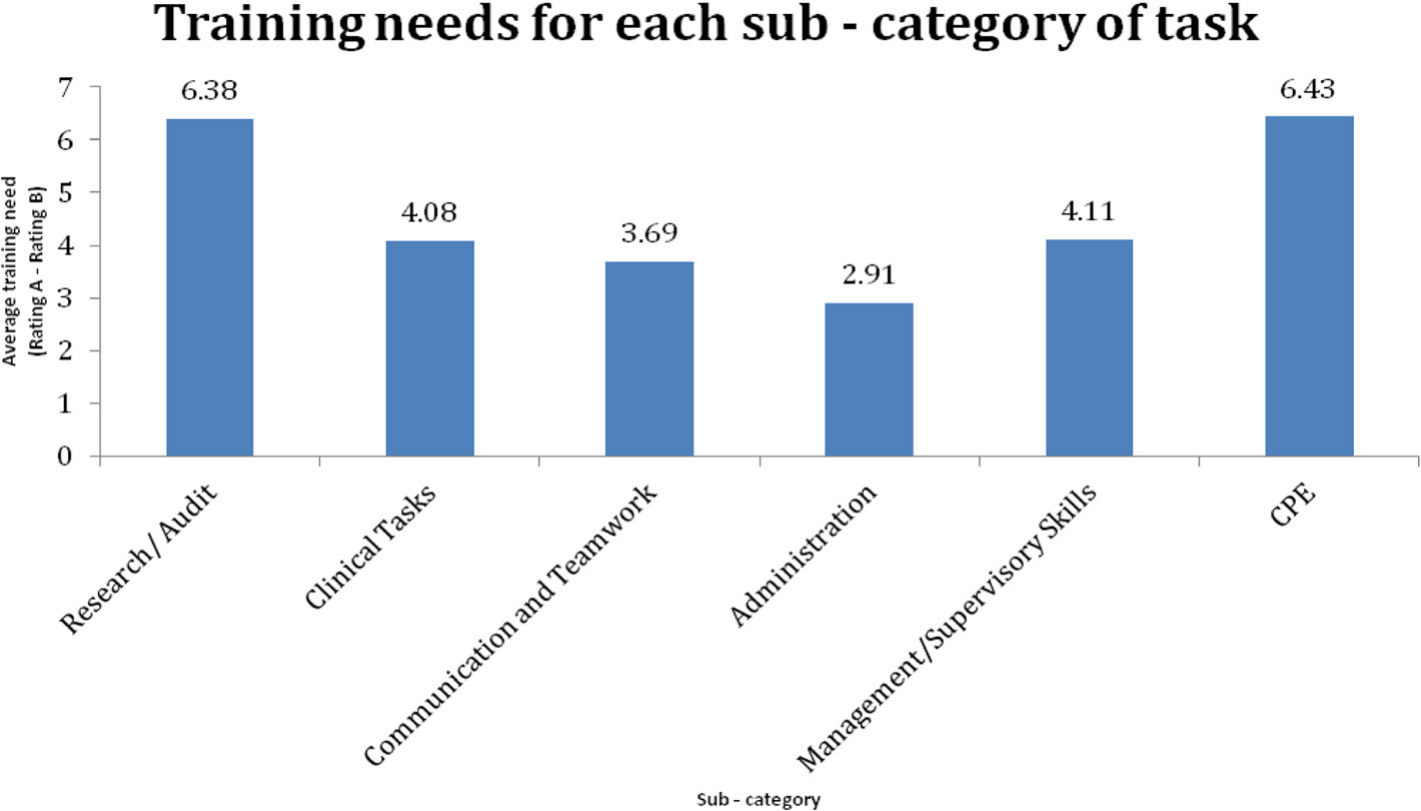
Training needs for subcategories of task

Figure 1 shows the relative training needs for each of the six task subcategories. This was derived by calculating the average difference score for the items in each of the given subcategories. The extent of need was calculated as the difference between the importance attributed to a task or item (Rating A on the questionnaire) and how well the respondents considered to have performed the task (Rating B on the questionnaire). The largest gap indicates the maximum training need. High scores on importance and low scores on performance indicate a training need. The higher the difference score, the greater is the training need. One can rank the training needs by the magnitude of these differences.

CPE had the highest average difference score of 6.43. This was closely followed by research/audit activities, with an average score of 6.38; management/supervisory skills, 4.11; clinical tasks, 4.08; communication and teamwork, 3.69; and administration, 2.91.

Table 2 lists the top 20 tasks in terms of the identified needs. The maximum training was required in the task of communication skills.

**Table 2:** Needs expressed by the respondents

Rank	Required needs	% of responses
1	Communication skills	12
2	Clinical skills	9
3	Administrative/Supervisory/Management	9
4	Research methods	9
5	Computer skills	7
6	Specialized training for related department	6
7	Emergency nursing	5
8	Treatment of intensive care patients (Adult and neonate)	5
9	Personnel management	5
10	Time management	5
11	Proper documentation	4
12	Public health/health promotion	4
13	Use of technical equipment	4
14	ECG (reading)	3
15	Disaster management	3
16	Health information management	3
17	Stress management	3
18	Counseling	2
19	Treatment of patients with mental health needs	2
20	Ultrasonology	2

## Discussion

It is not entirely clear how a health care professional would determine which tasks are crucial and how well they perceive their actual performance of that task to determine the need. However, these may be influenced by several factors, including their motivation to continue learning, a special interest in that particular task, an encounter or deficiency in their previous education, and their satisfaction or dissatisfaction with the management of the department among others. Further research is warranted to investigate the motivation for determining the need.

The respondents indicated various needs in the section of the questionnaire that had questions on needs they considered necessary. Effective communication, clinical skills, management, research methods, computer training, health promotion, disaster management stress and time management, and proper documentation, among others, were the most commonly reported needs.

Most respondents required clinical skill training and training associated with the department in which they worked. This suggests that they would prefer any type of training that concentrates on the current advanced practices in the department they have been assigned. Clear and appropriate communication and interdisciplinary collaboration is critical for delivering quality care for complex patients in the present health care settings [20]. Collaborative practice among all health care professionals creates a positive work environment. Poor communication and a lack of teamwork or collaboration have been persistent problems in health care.

Most of our findings are similar to those of other studies. However, research/audit activities was ranked second. By contrast, a previous study [15] revealed a perceived lack of such type of need among nurses. Moreover, the previous study indicated that research activities were required by other health care professionals. In contrast to other study results, modern nurses might be more receptive to the idea of research needs in nursing. Although research is not extensively conducted in developing countries, research within a local context to improve health care quality has gained increasing attention in Saint Lucia. The MOH has undertaken a project to publish a research agenda, which will follow the Caribbean Research Council’s research agenda for health.

The only full-fledged health care teaching institution in Saint Lucia is the Sir Arthur Lewis Community College, Department of Health Science—General Nursing; it offers a three-year Associate Degree in the General Nursing program. At the end of the training, the students receive an Associate Degree in Nursing and become eligible for the regional licensing exam to become a registered nurse. This would account for most respondents having attained at least an associate degree.

Although ranked 19th among the top 20 needs expressed by the respondents, considering the mental health reform in Saint Lucia, there exists a crucial need for further training in the management and care of patients with mental health conditions. A mental health care facility focusing on promoting mental wellness has been recently established. The staff of other health care facilities also require training in mental health to help with detecting and referring patients with mental problems. This also demonstrates how the employers and employees might have different views regarding needs.

Only by addressing the training needs of health care personnel can we hope to achieve the millennium development goals. Providing training according to needs is vital, particularly in developing countries where resources are limited. The needs of health care personnel are variable; every worker has unique needs. Their perceived and actual needs change with time, place, and clinical caseload. Moreover, needs vary according to the environment in which the professional practices and the resources that are available to perform various tasks.

At present, need assessment is crucial because of the volume of health care projects being undertaken in Saint Lucia. Our findings should inform the MOH about the needs of the proposed research agenda and can be used for decision making regarding transition to the new national hospital and for other health care institutions during transitioning. This study is a prime example of evidence-based decision making, which is lacking in the health sector of developing countries.

The assessment provides empirical data to agencies interested in contributing to the development of health care in Saint Lucia, such that they can provide assistance where required. It is particularly true at present with the pending commissioning of the new national hospital in Saint Lucia. This assessment can form the basis for training across various spectrums as an attempt to empower the workforce in a manner it has never been empowered before: training needs identified by themselves.

This study only involved the employees not the employers. It was observed that involving potential trainees and their employing institutions in needs assessment might help the designers of a training program to focus on priorities relevant to the local context [21]. Thus, this is the major limitation of our study and warrants further research.

## Conclusions

In this study, CPE was found to be the most required among health care personnel in Saint Lucia, followed by training in research. Communication skills accounted for the biggest need. The present findings can be used as a basis for implementing such programs. The authorities responsible for providing continuous training for various disciplines must plan and execute programs that meet the needs of the people they represent.

Providing training according to needs is vital, particularly in developing countries where resources are extremely limited. The present research methodology and findings also offer perspective on how to conduct needs assessment and serve as a reference for developing countries whose health care environments are similar to that in Saint Lucia.

### Ethics approval and consent to participate

Ethical approval was obtained from the Saint Lucia Medical and Dental Council on August 9, 2011. Participation was voluntary and individually written consent was not required.

### Consent for publication

Not applicable. This article does not contain any individual person’s data.

### Availability of data and materials

The dataset supporting the conclusions of this article is included within the article as Additional file 2.

## Additional files

Additional file 1: Training Needs Assessment Questionnaire. (DOCX 40kb) Additional file 2: The dataset supporting the conclusions of this article. (XLS 441kb)

## Abbreviations

CE: continuing education
CPE: continuing professional education
MOH: ministry of health
