# Supplementary material for: Training needs assessment of health care professionals in a developing country: the example of Saint Lucia
Source: BMC Med Educ. 2016 Apr 16;16:112. doi: 10.1186/s12909-016-0638-9 (PMC4847227; doi:10.1186/s12909-016-0638-9)
Supplement: Additional file 1: — Training Needs Assessment Questionnaire. (DOCX 40kb) [file 12909_2016_638_MOESM1_ESM.docx]

To what extent could appropriate training raise your performance on this activity? Please rate anticipated performance following training ALONE. 1 = little or no improvement; 7 = great improvement.

Rating of 1-7 no scope at all = 1; great scope = 7

How important is the activity to the successful performance of your job?

Rating 1-7 not at all important=1; very important = 7

How well do you consider that you currently perform this activity?

Rating of 1-7 not well= 1; very well = 7

To what extent could appropriate organisational changes raise your performance on this activity? Please rate anticipated performance following organisational change ALONE. 1= little or no improvement; 7 = great improvement.

Rating of 1-7 no scope at all = 1; great scope = 7

|  | **A** | **B** | **C** | **D** |
| --- | --- | --- | --- | --- |
| **1. Establishing a relationship with patients** |  |  |  |  |
| **2. Inputting accurate data in written or computerized records/or routine data input.** |  |  |  |  |
| **3. Conducting any kind of research** |  |  |  |  |
| **4. Appraising your own performance** |  |  |  |  |
| **5. Getting on with your colleagues in all disciplines** |  |  |  |  |
| **6. Communicating with patients effectively** |  |  |  |  |
| **7. Identifying areas of practice that should be investigated** |  |  |  |  |
| **8. Treatment of patients** |  |  |  |  |
| **9. Introducing new ideas at work** |  |  |  |  |
| **10. Accessing relevant literature for your clinical work** |  |  |  |  |
| **11. Providing feedback to colleagues working in all disciplines** |  |  |  |  |
| **12. Giving adequate information to patients and their families at all times** |  |  |  |  |
| **13. Benefit of journal subscription to your development** |  |  |  |  |
| **14. Instructing or sharing new knowledge with colleagues and/or students about new practices or procedures** |  |  |  |  |
| **15. Planning and organizing an individual patient’s care** |  |  |  |  |
| **16. Organizing your own time effectively** |  |  |  |  |
| **17. Using technical equipment, including computers for everyday execution of your duties** |  |  |  |  |
| **18. Undertaking health promotion activities** |  |  |  |  |
| **19. Making do with limited resources** |  |  |  |  |
| **20. Assessing patients’ clinical needs** |  |  |  |  |
| **21. Designing a research study** |  |  |  |  |
| **22. Working as a member of an inter-disciplinary team** |  |  |  |  |
| **23. Accessing research resources (e.g. time, money, information, equipment)** |  |  |  |  |
| **24. Undertaking administrative duties** |  |  |  |  |
| **25. Personally coping with change in the health service** |  |  |  |  |
| **26. Benefit of CPE toy our job.** |  |  |  |  |
| **27. Mandatory CE for re-licensure** |  |  |  |  |
| **28. Benefit of journal subscription to your development** |  |  |  |  |
| **29. Frequency of CE** |  |  |  |  |
| **30. Access to CPE sessions** |  |  |  |  |

**SECTION 2: Specific training needs**

**Please specify the areas of your job in which you would like to receive further training or instruction. List these in order of importance:**

**1.**

**2.**

**3.**

**4.**

**5.**
